# Homeostase do Tiol na Doença Cardíaca Reumática: Biomarcador ou Fator de Risco?

**DOI:** 10.36660/abc.20210693

**Published:** 2021-09-01

**Affiliations:** 1 Faculdade de Medicina da Universidade de São Paulo Instituto do Coração Unidade Clínica de Doenças Valvares São Paulo SP Brasil Unidade Clínica de Doenças Valvares - Instituto do Coração - Hospital das Clínicas da Faculdade de Medicina da Universidade de São Paulo, São Paulo, SP – Brasil

**Keywords:** Febre Reumática, Doenças Cardiovasculares, Homeostase, Sulfidrila, Fatores de Risco, Biomarcadores

As lesões da febre reumática e da doença cardíaca reumática (DCR) resultam de uma complexa rede de vários genes e proteínas que controlam respostas imunes inatas e adaptáveis após uma faringotonsilite por Streptococcus pyogenes. O processo inflamatório subsequente leva ao desenvolvimento de lesões cardíacas com alta produção de citocinas inflamatórias conduzindo à calcificação e à fibrose valvar.^1^ No entanto, as vias celulares além desses fenômenos imunoregulados não foram totalmente elucidadas.

O estresse oxidativo é uma condição biológica marcada por um desequilíbrio entre a produção de espécies reativas de oxigênio e sua redução.^2^ Os distúrbios nesse equilíbrio redox podem causar a superprodução de peróxidos e radicais livres que danificam todos os componentes celulares, incluindo proteínas, lipídios e DNA.^3^

O estresse oxidativo excessivo também desempenha um papel importante na patogênese de doenças autoimunes, aumentando a inflamação e modificando a tolerância imunológica.^4^ Há uma complexa relação recíproca entre estresse oxidativo, apoptose e autofagia. Isso é especialmente relevante no contexto das doenças autoimunes.^5^ O papel do estresse oxidativo na DCR ainda é desconhecido.

O tiol ou sulfidrilo (–SH) é uma forma altamente ativa e versátil de enxofre reduzido em biomoléculas. Está presente em aminoácidos como a cisteína (Cis), em peptídeos e proteínas e é particularmente sensível às reações redox.^6^ Eles podem atuar como um sensor redox crucial, bem como um interruptor capaz de modificar a função proteica e a interatividade pós-traducional. O tiol proteoma e tiol-oxidorredutases são áreas emergentes de investigação. As alterações no estado de tiol-dissulfeto redox têm sido estudadas em diferentes doenças, como câncer, neurodegenerativa e cardiovascular.^7^

Os processos oxidativos podem converter os tióis em muitas moléculas diferentes. O tiol-dissulfeto é um dos produtos de reações oxidativas nas quais os tióis estão envolvidos. Estudos atuais demonstraram que a razão tiol-dissulfeto pode ser de valor significativo como um promissor marcador de estresse oxidativo.^8^

O estudo do nível dos tióis séricos e da homeostase tiol-dissulfeto analisou os níveis de tiol em pacientes com DCR e indivíduos saudáveis. Os autores mostraram uma correlação positiva entre os níveis de dissulfeto e a gravidade da estenose mitral, bem como entre a relação dissulfeto/tiol total e nativa com a pressão da artéria pulmonar, o diâmetro do átrio esquerdo e a gravidade da estenose mitral. Os autores concluem que os níveis plasmáticos de tióis foram significativamente mais baixos em pacientes com doença valvar mitral (DVM) em comparação com o grupo controle. Os níveis de dissulfeto e a razão dissulfeto/tiol foram maiores em pacientes com DVM.^9^

Dada a complexidade e múltiplos compartimentos de toda a organização corporal, o termo “estresse oxidativo orgânico” pode ser inapropriado. Além disso, a associação entre as mudanças de tiol redox está provavelmente associada a fatores sistêmicos, como disfunção endotelial, devido à inflamação relacionada ao lipídio, diabetes, tendência trombótica sistêmica e outros fatores desconhecidos. Nesse sentido, a questão é se as mudanças oxidativas plasmáticas refletem um biomarcador do próprio processo de doença localizada em vez de um fator de risco para doenças vasculares e cardíacas.^10^

De qualquer forma, o estudo destacou uma nova área de investigação dos pools de tióis plasmáticos em DCR, trazendo informações relevantes na fisiopatologia, estágio da doença e até prognóstico. Mais estudos em valvopatia são necessários para elucidar o mecanismo fisiopatológico neste órgão-alvo. Posteriormente, pode ser possível correlacioná-los com os achados no plasma para finalmente obter biomarcadores específicos.
