# Supplementary material for: Homeostase do Tiol na Doença Cardíaca Reumática: Biomarcador ou Fator de Risco?
Source: Arq Bras Cardiol. 2021 Sep 1;117(3):444–5. [Article in Portuguese] doi: 10.36660/abc.20210693 (PMC8462955; doi:10.36660/abc.20210693)
Supplement: Translation

# Translation: Thiol Homeostasis in Rheumatic Heart Disease: Biomarker or Risk Factor?

**Affiliations:** 1 São Paulo SP Brazil Unidade Clínica de Doenças Valvares - Instituto do Coração - Hospital das Clínicas da Faculdade de Medicina da Universidade de São Paulo, São Paulo, SP – Brazil

**Keywords:** Rheumatic Fever, Cardiovascular Diseases, Homeostasis, Sulphydril, Risk Factors, Biomarkers

Rheumatic fever and rheumatic heart disease (RHD) lesions result from a complex network of several genes and proteins that control innate and adaptive immune responses following a Streptococcus pyogenes pharyngo-tonsillitis. The succeeding inflammatory process leads to the development of cardiac lesions with high production of inflammatory cytokines leading to calcification and valvular fibrosis.^1^ However, the cellular pathways beyond these immunoregulated phenomena have not been fully elucidated.

Oxidative stress is a biological condition marked by an imbalance between reactive oxygen species production and its reduction.^2^ Disturbances in this redox balance can cause the overproduction of peroxides and free radicals that damage all cellular components, including proteins, lipids and DNA.^3^

Excessive oxidative stress also plays an important role in autoimmune diseases pathogenesis, increasing inflammation and modifying the immune tolerance.^4^ There is a complex reciprocal relationship between oxidative stress, apoptosis and autophagy. This is especially relevant in the context of autoimmune disorders.^5^ The role of oxidative stress in RHD is still unknown.

Thiol or sulphydryl (–SH) is a highly active and versatile form of reduced sulfur in biomolecules. It is present in amino acids such as cysteine (Cys) in peptides and proteins and is particularly sensitive to redox reactions.^6^ They may act as a crucial redox sensor as well as a switch able to modify protein function and interactivity post-translationally. The thiol proteome and thiol-oxidoreductases are emerging areas of investigation. Changes in thiol-disulfide redox state have been studied in different diseases such as cancer, neurodegenerative and cardiovascular.^7^

Oxidative processes can convert thiols into many different molecules. Thiol-disulfide is one of the products of oxidative reactions in which thiols are involved. Current studies demonstrated that the thiol-disulfide ratio might be of significant value as a promising oxidative stress marker.^8^

The study of serum thiols level and thiol-disulfide homeostasis accessed thiol levels in patients with RHD and healthy subjects. The authors showed a positive correlation between disulfide levels and the severity of mitral stenosis as well as between total and native disulfide/thiol ratio with pulmonary artery pressure, left atrium diameter, and mitral stenosis severity. The authors conclude that plasma thiol levels were significantly lower in patients with mitral valve disease (MVD) compared to the control group. Disulfide levels and the disulfide/thiol ratio were higher in patients with MVD.^9^

Given the complexity and multiple compartments of whole body organization, the term “organismal oxidative stress” can be inappropriate. Furthermore, the association between thiol redox shifts is likely associated with systemic factors, such as endothelial dysfunction, due to lipid-related inflammation, diabetes, systemic thrombotic tendency, and other unknown factors. In this regard, the question is if plasma oxidative shifts reflect a biomarker of the localized disease process itself rather than a risk factor for vascular and heart disease.^10^

In any case, the study highlighted a new investigation area of plasma thiols pools in RHD, bringing relevant information in the pathophysiology, disease stage and even prognosis. Further studies in valvular heart disease are needed to elucidate the pathophysiological mechanism in this target organ. Subsequently, it may be possible to correlate them with findings in plasma to obtain specific biomarkers finally.
